# Neoadjuvant Chemotherapy Followed by Interval Debulking for Advanced-Stage Endometrial Cancer: Survival Outcome Based on Surgical and Molecular Characteristics

**DOI:** 10.3390/curroncol33080448

**Published:** 2026-07-27

**Authors:** Mira Kheil, Tariq Mekkaoui, Emily Andresan, Gloria Fung, Madison Miller, Jamie G. Joseph, Anqi Wang, Ali Al Asadi, Mohamed Elshaikh, Sarfraz Ahmad, Ahmad Awada

**Affiliations:** 1 Department of Obstetrics & Gynecology, Henry Ford Health, Detroit, MI 48202, USA; mkheil1@hfhs.org (M.K.); tmekkao1@hfhs.org (T.M.); 2 School of Medicine, Michigan State University, Detroit, MI 48201, USA; eandres1@hfhs.org; 3 School of Medicine, Wayne State University, Detroit, MI 48201, USA; gfung1@hfhs.org; 4 Department of Gynecologic Oncology, New York Presbyterian/Columbia University Irving Medical Center, New York, NY 10032, USA; mem2281@cumc.columbia.edu; 5 Department of Public Health & Epidemiology, Henry Ford Health, Detroit, MI 48202, USA; jjosep11@hfhs.org (J.G.J.); awang7@hfhs.org (A.W.); 6 Department of Radiation Oncology, Henry Ford Health, Detroit, MI 48202, USAmelshai1@hfhs.org (M.E.); 7 Gynecologic Oncology Program, AdventHealth Cancer Institute, Orlando, FL 32804, USA; 8 Department of Gynecologic Oncology, Henry Ford Health, Detroit, MI 48202, USA

**Keywords:** endometrial cancer, advanced-stage disease, neoadjuvant chemotherapy, interval debulking surgery, surgical features, molecular characterization, clinicopathological factors, survival analysis

## Abstract

Objective: To examine survival outcomes and identify clinicopathological factors associated with survival in patients with advanced-stage endometrial cancer who received neoadjuvant chemotherapy before interval debulking surgery (NACT-IDS). Methods: A single-center retrospective cohort study was conducted of patients who were diagnosed with advanced-stage (2009 FIGO IIIB, IIIC, IV) endometrial cancer (2012–2024) and underwent NACT-IDS. Tumor response to NACT was determined with computed tomography and the RECIST criteria, and demographic, clinicopathologic, perioperative, and tumor molecular features (mismatch repair protein [MMR] status and p53 pattern) were collected from medical chart review. Association between tumor molecular features and response to NACT was determined. Primary endpoints were progression-free and overall survival, analyzed with univariate Cox and stratified Kaplan–Meier analysis. Results: Of 42 consecutive patients (median age of 68 years), the majority (n = 26; 61.9%) had a partial tumor response to NACT, with only five (11.9%) having a complete response, four (9.5%) having stable disease, and seven (16.7%) having progressive disease. Most cases had no residual tumor after IDS (n = 35; 83.3%). MMR protein status was associated with the tumor response to NACT (*p* = 0.013), but p53 status was not. During follow-up, 24 patients died (57.1%), and 31 (73.8%) died or had disease progression. Tumor response to NACT and resection margin status were associated with progression-free survival in unadjusted analyses, but no covariate adjustment was possible with limited sample size. Conclusions: This study highlights MMR-deficiency, response to NACT, and surgical resection status as clinicopathologic features of interest for future studies of prognostic factors and alternative therapies in advanced-stage endometrial cancer.

## Simple Summary

Advanced-stage endometrial cancer is difficult to treat because many patients have disease that cannot be safely removed with surgery at the time of diagnosis. In these situations, chemotherapy may be given first to shrink the cancer before surgery, an approach called neoadjuvant chemotherapy. However, it is still unclear which patients benefit most from this strategy and which factors influence their outcomes. In this study, we reviewed the medical records of 42 patients with advanced-stage endometrial cancer who received neoadjuvant chemotherapy followed by interval debulking surgery. Most patients experienced tumor shrinkage after chemotherapy, and more than 80% had no visible tumor remaining after surgery. Although no individual clinical or molecular factor independently predicted survival after statistical adjustment, tumor response to chemotherapy and the complete surgical removal of the tumor showed potential associations with progression-free survival. These findings add to the growing evidence supporting neoadjuvant chemotherapy as a treatment option for selected patients with advanced endometrial cancer and highlight the need for larger studies to better identify which patients are most likely to benefit from this approach.

## 1. Introduction

Endometrial cancer is the fourth most common cancer affecting women in the United States [1]. While patients diagnosed with early-stage endometrial cancer generally have favorable outcomes, with 5-year overall survival rates of approximately 83% to 99% [2], patients diagnosed with advanced-stage disease have substantially poorer prognoses [3], with 5-year mortality rates approaching 42% to 71% [4]. In fact, patients with stage IVB disease generally have very poor overall survival (OS) of only 12 to 24 months, even after receiving multimodal treatment [5,6], underscoring the need for more effective treatment approaches for patients with advanced-stage disease.

Therapeutic strategies for endometrial cancer have evolved alongside refinements in histologic classification, advances in molecular profiling, and shifts in staging and adjuvant treatment paradigms [3,7]. The current standard-of-care for advanced-stage endometrial cancer is primary cytoreductive surgery (PCS) [8], which reduces tumor burden and is associated with improved survival [3,5]. However, PCS is not often feasible for patients who have advanced-stage disease, extra-abdominal metastasis, unresectable intra-abdominal disease, or certain medical comorbidities. Overall, the lack of evidence-based therapies for advanced-stage disease, particularly for patients who are ineligible for surgery, highlights the need for large-scale studies to assess whether alternative treatment strategies can meaningfully improve survival outcomes [9].

Neoadjuvant chemotherapy followed by interval debulking surgery (NACT-IDS) is an emerging alternative therapy for patients with endometrial cancer who may be ineligible for primary PCS due to advanced disease and extensive transperitoneal involvement [6,10,11]; however, despite growing use and encouraging evidence, robust clinical trials comparing NACT-IDS to PCS are lacking [12,13,14]. Additionally, optimal patient selection criteria for NACT-IDS and the key clinicopathologic predictors of survival in patients with unresectable advanced disease are lacking and remain undefined [13].

Within this context, determining the real-world effectiveness of NACT-IDS and the patient features needed for accurate risk stratification will be essential for improving survival outcomes in patients with advanced-stage, unresectable endometrial cancer. Therefore, the primary aim of this retrospective cohort study was to determine the clinicopathological factors associated with survival outcomes in patients with advanced endometrial cancer who underwent NACT-IDS. We characterized NACT tumor responses, surgical cytoreductive outcomes, and survival to begin clarifying the role of NACT in advanced endometrial cancer and identify factors that might inform and improve treatment decision-making.

## 2. Methods

We conducted a retrospective cohort study of patients with advanced-stage endometrial cancer who underwent NACT-IDS between January 2012 and January 2024 at a single urban institution. The decision to proceed with NACT prior to surgery was made due to concern for inoperable disease on either imaging or diagnostic laparoscopy. All patients received 3 to 6 cycles of platinum-based and taxane chemotherapy before surgery. Patients who had radiation therapy before surgery were excluded. This study was approved by the Henry Ford Health Institutional Review Board (IRB#18311, Approval date: 10 March 2025). The requirement for informed consent was waived due to the retrospective nature of the study.

The following demographic and clinicopathological features were collected from the medical record: age at diagnosis, race, body mass index (BMI), tumor biopsy histologic subtype (carcinosarcoma, clear cell carcinoma, endometrioid, mixed, serous, and undifferentiated), tumor biopsy grade, 2009 International Federation of Gynecology and Obstetrics (FIGO) stage at diagnosis (IIIB, IIIC, or IVB), and levels of the serum tumor marker cancer antigen–125 (CA-125) at time of diagnosis. FIGO criteria were used for endometrial tumor biopsy grading. Tumor grades were categorized as grade 1 (well-differentiated), grade 2 (moderately differentiated), or grade 3 (poorly differentiated). Only patients with FIGO cancer stages IIIB, IIIC, or IVB were included in this study analysis.

The following therapeutic responses, surgical features, surgical outcomes, and tumor molecular features were also extracted: response to preoperative NACT, surgical approach (open or robotic), postoperative resection margin status (R0, R1, and R2), lymphovascular space invasion (LSVI), resected tumor mismatch repair (MMR) protein status (deficient or proficient), and resected tumor p53 immunohistochemistry (IHC) pattern (aberrant or wild type). Tumor response to preoperative NACT was assessed with computed tomography (CT) according to the revised RECIST (Response Evaluation Criteria in Solid Tumors) criteria, a standardized imaging-based classification system [15]. Responses included complete response (CR, no remaining lesions); partial response (PR, ≥30% decrease in total target lesion diameters); stable disease (SD, no tumor reduction or decrease); and progressive disease (PD, ≥20% increase in target lesion diameter). Postoperative resection margin status was defined as R0 (no residual tumor), R1 (microscopic residual tumor), and R2 (macroscopic residual tumor). Treatment and pathologic characteristics for the subgroup of patients who had a complete or partial response to NACT were gathered for descriptive purposes, including number of NACT cycles, use of neoadjuvant immunotherapy (IO), LSVI, pathology analysis confirmation of complete response, and cancer recurrence during follow-up.

The association of MMR status and p53 pattern with the NACT tumor response was analyzed with Fisher’s exact test with the Freeman-Halton extension for contingency tables larger than 2 × 2. The primary outcomes were OS and progression-free survival (PFS). The OS was defined as the time from diagnosis to the time of last follow-up or death from any cause. The PFS was defined as the time from initiation of treatment to the time of radiographic disease progression demonstrated by CT or death from any cause, whichever came first. Univariate Cox models were used to determine the association of clinical and therapeutic features with OS and PFS during the study period. Clinical and therapeutic features analyzed included cancer stage at diagnosis, NACT tumor response, surgical approach, postoperative resection margin status, resected tumor MMR status, and resected tumor p53 pattern. For each Cox model, *p*-values from the likelihood ratio test were obtained to examine the associations between the variables of interest and survival outcomes. Departures from the proportional hazard assumptions required for the Cox models, along with small cell counts and potential unmeasured confounding, limited inferential conclusions; therefore, findings should be interpreted as largely descriptive.

Time-to-survival outcomes (OS and PFS) at 1 and 5 years were estimated with the Kaplan–Meier method. Kaplan–Meier estimated survival probabilities were determined and reported with 95% confidence intervals (CI). Kaplan–Meier analyses were stratified by cancer stage at diagnosis, postoperative margin status, p53 pattern, and MMR protein status. Postoperative margin status was stratified as R0 versus R1/R2 for univariate Cox and Kaplan–Meier analyses. Statistical significance was set at *p* < 0.05. All analyses were done in R version 4.4.0.

## 3. Results

### 3.1. Patient Demographics, Treatment, and Tumor Characteristics

A total of 42 consecutive patients with advanced-stage endometrial cancer who underwent NACT-IDS were included. In the neoadjuvant setting, all patients received Platinum and taxane-based chemotherapy. Additionally, two patients received Adriamycin, one received Bevacizumab, one received Trastuzumab, and two received Dostarlimab. Time elapsed between the end of NACT and surgery ranged from one to three months. In the adjuvant setting, two patients passed shortly after surgery. Patients with partial response and suboptimal cytoreduction received adjuvant chemotherapy with Carboplatin and Taxol. A total of 11 patients also received adjuvant pembrolizumab and lenvatimib; 2 were pMMR, 6 were dMMR, and 3 had missing MMR. A total of 12 patients underwent adjuvant radiation therapy (RT).

There were 19 Black patients (45%), 23 White patients (55%), and the median age at diagnosis was 68 years (IQR, 63–72). The median BMI was 32.3 kg/m^2^ (IQR 27.6–38.3). Of the various tumor biopsy histological subtypes, serous was the most common (n = 20; 47.6%), followed by endometrioid (n = 12; 28.6%). Most tumors were grade 3 (n = 33; 78.6%). There was only 1 patient (2.4%) with stage IIIB disease, whereas 11 (26.2%) had stage IIIC and 30 (71.4%) had stage IVB disease. Of the 37 patients who had CA-125 determined at diagnosis, the median level was 117 U/mL (IQR, 27–580; n = 37) (Table 1).

The tumor response to NACT as assessed by the RECIST criteria showed that most patients had a partial response (n = 26; 61.9%), while five (11.9%) had a complete response, four (9.5%) had stable disease, and seven (16.7%) had progressive disease. Open surgery was performed for most patients (n = 33; 78.6%), with only nine patients (21.4%) undergoing robotic surgery. After debulking surgery, most patients (n = 35; 83.3%) had R0 tumor margin status, indicating no residual tumor. There were five patients (11.9%) who had R1 margin status, indicating microscopic residual tumor, and two patients (4.8%) had R2 margin status, indicating macroscopic residual tumor. Postoperative LVSI was observed in a little more than half of patients (n = 22; 52.4%). Of the tumors that were postoperatively evaluated for MMR protein status and p53 patterns, most (n = 23/29; 79.3%) were MMR deficient, and a little over half (n = 16/31; 51.6%) had aberrant p53 (Table 2).

Patients who had a complete (n = 5) or partial (n = 26) response to NACT had a variety of treatment and pathologic characteristics. Most patients in both groups had undergone 4 to 6 cycles of NACT. There were two patients in the partial response group who underwent neoadjuvant IO therapies. None of the patients with a complete response had disease with LVSI, whereas more than half of the patients in the partial response group did (n = 14/26; 53.8%). There were three total patients, two in the complete response group and one in the partial response group, for whom final pathology analysis showed a complete response. Whereas only one patient (20%) in the complete response group had recurrent disease during follow-up, 20 of the 26 patients in the partial response group (76.9%) had disease recurrence (Table 3). These results should be interpreted with caution due to extremely low/zero cell counts and should be used for purely exploratory and hypothesis-generating purposes.

### 3.2. Molecular Status and Tumor Response to NACT

Tumor MMR protein status was significantly associated with the tumor response to NACT (*p* = 0.013). Of the 23 patients with MMR-deficient tumors, most (n = 17; 73.9%) had a partial response. For the six patients with MMR-proficient tumors, none had a complete response, whereas two patients each (33.3%) had a partial response, stable disease, and progressive disease. Tumor p53 patterns were not associated with the tumor response to NACT (*p* = 0.365) (Table 4).

### 3.3. Survival Outcomes

**Figure 1:**
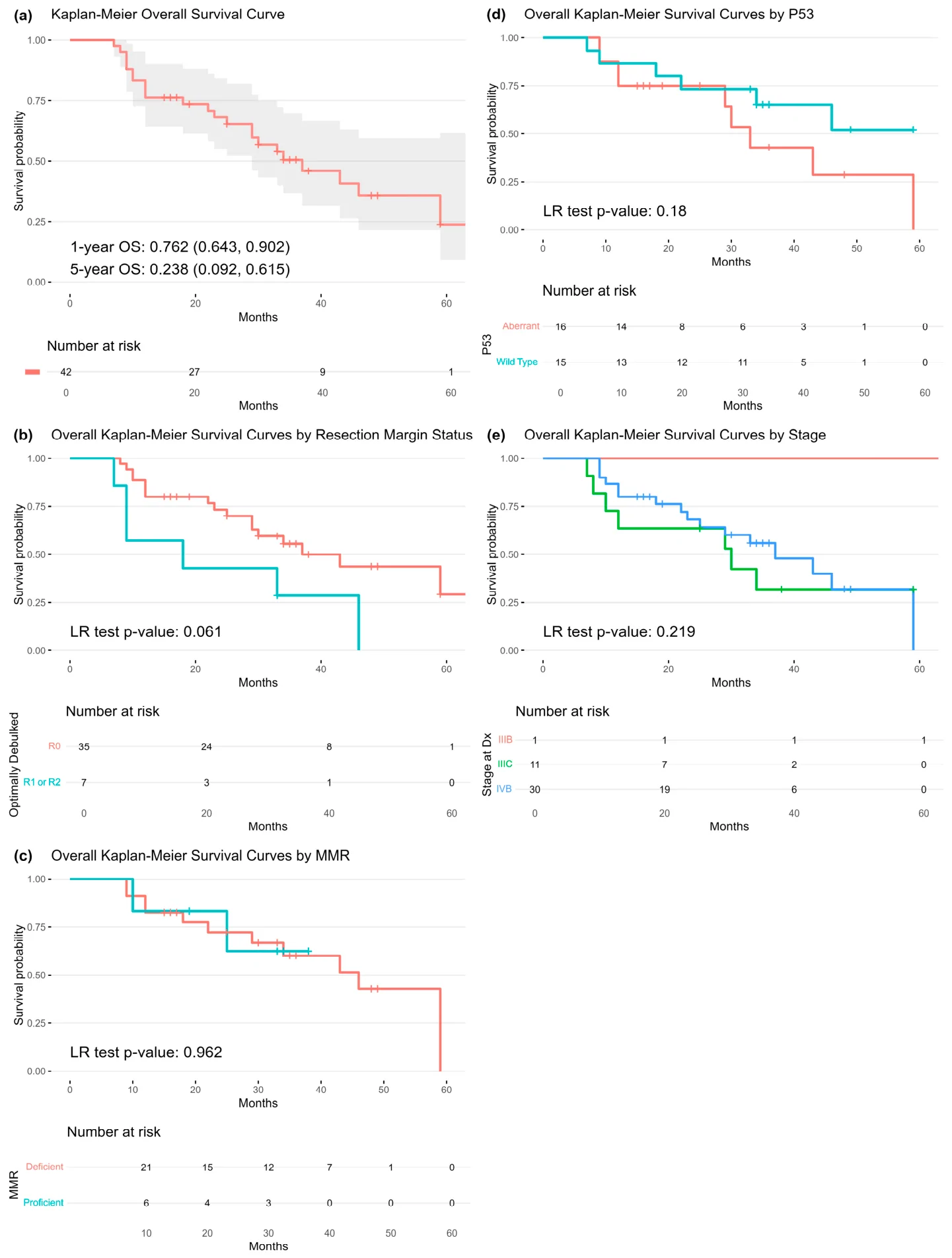
Overall survival analyses by Kaplan–Meier analysis method stratified by (**a**) total population, (**b**) cancer stage at diagnosis, (**c**) postoperative/resection margin status, (**d**) MMR protein status, and (**e**) p53 pattern/status. Abbreviations: OS, overall survival; CI, confidence interval; MMR, mismatch repair.

**Figure 2:**
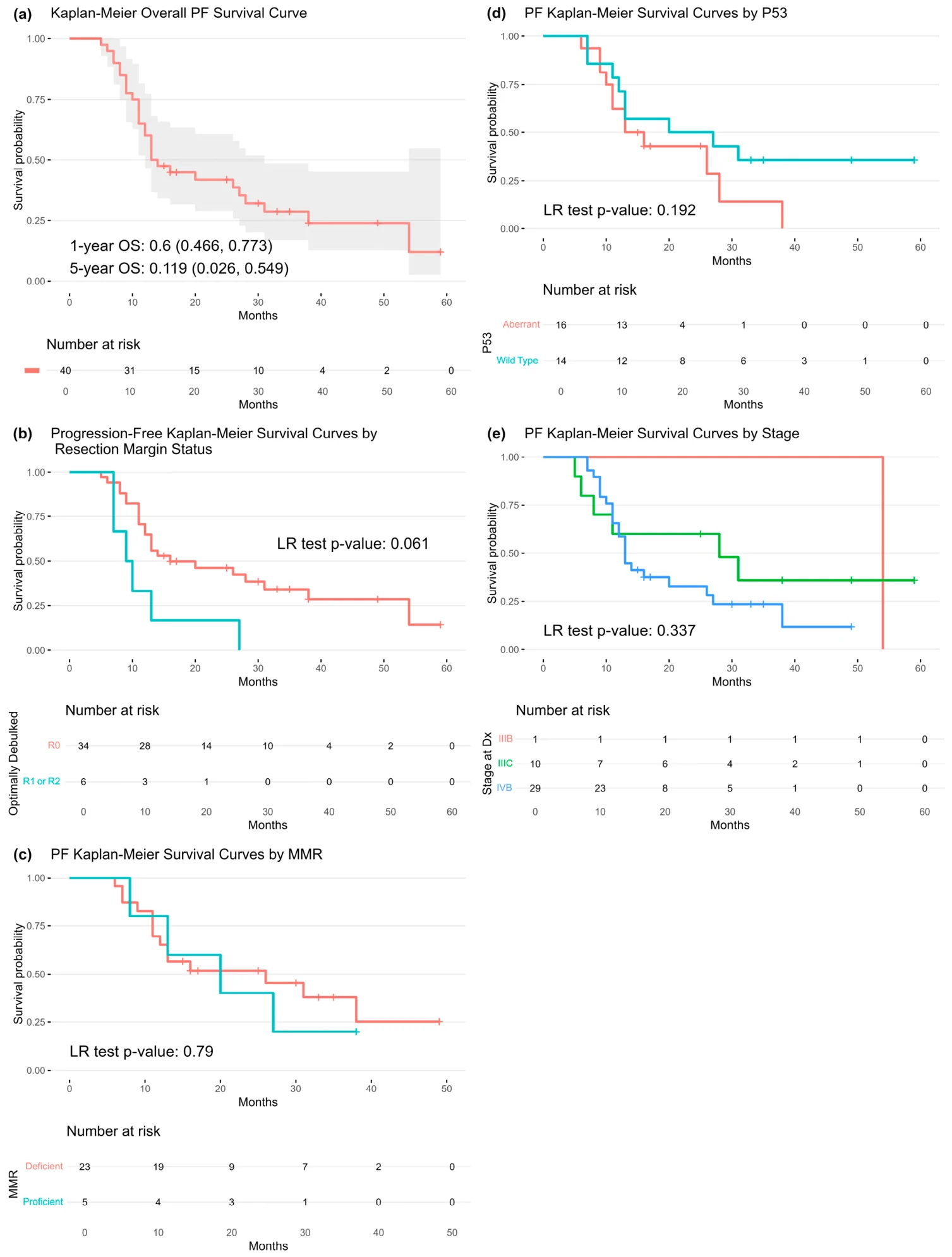
Progression-free survival analyses by Kaplan–Meier analysis method stratified by (**a**) total population, (**b**) cancer stage at diagnosis, (**c**) postoperative/resection margin status, (**d**) MMR protein status, and (**e**) p53 pattern/status. Abbreviations: PFS, progression-free survival; CI, confidence interval; MMR, mismatch repair.

During the study period, 24 patients (57.1%) died. The estimated overall survival probability was 0.81 (95% CI, 0.699–0.937) at 1 year and 0.24 (95% CI, 0.092–0.614) at 5 years (Figure 1). Also, during the study period, 31 patients (73.8%) died or had disease progression. The estimated probability of progression-free survival was 0.762 (95% CI, 0.643–0.902) at 1 year and 0.238 (95% CI, 0.092–0.615) at 5 years (Figure 2). The OS and PFS curves stratified by cancer stage at diagnosis, resection margin status, MMR protein status, and p53 pattern are also included in Figure 1 and Figure 2. The tumor response to NACT and resection margin status were both significantly associated with PFS in Cox analyses, suggesting a need for more investigation (both *p* = 0.04) (Table 5).

## 4. Discussion

In this retrospective cohort study, we comprehensively characterized the clinicopathologic, perioperative, and molecular features of a group of patients with advanced endometrial cancer who underwent the alternative/emerging treatment strategy of NACT followed by IDS. Estimates of survival in this group showed that although the probabilities of both OS and PFS at one year were favorable (0.81 (95% CI, 0.699–0.937) and 0.762 (95% CI, 0.643–0.902) respectively), survival probabilities at five years were low (0.24 (95% CI, 0.092–0.614) and 0.238 (95% CI, 0.092–0.615), respectively).

Notably, more than 60% of patients showed a partial response to NACT per the RECIST radiographic criteria, while only about 12% had a complete response and almost 17% had evidence of progressive disease. Also, MMR protein status was associated with the preoperative tumor response to NACT, in which most patients with MMR-deficient tumors had complete or partial responses. Although no clinicopathologic or other features were clearly associated with survival, NACT tumor response and resection margin status were significantly associated with PFS in Cox analyses. Associations found in this study should be interpreted with caution due to small sample sizes, multiple outcome models being run (12 total), and lack of adjustment for confounding due to limited degrees of freedom. Our results do suggest potential factors worth further exploration in larger controlled and real-world studies. Overall, this study adds descriptive context to the limited evidence on outcomes in patients with advanced-stage endometrial cancer who have undergone NACT-IDS.

NACT followed by IDS is emerging as a feasible treatment strategy for certain patients with advanced-stage endometrial cancer, particularly those who are not good candidates for PCS due to anticipated surgical complexity or morbidity risk [3,11,16,17]. By reducing tumor burden before surgical intervention, NACT may improve operability, increase the likelihood of complete gross resection [16,18], and offer a more tolerable initial approach for patients with high-volume or medically complex disease. Although optimal cytoreduction remains an important clinicopathologic determinant of survival in patients with advanced disease [6,19], other factors associated with favorable outcomes among patients who undergo NACT-IDS, as well as the prognostic significance of the tumor response to NACT, remain unknown [12].

Most patients in our study, over 80%, had complete gross tumor resections after NACT. Although complete resection was not significantly associated with improved survival, we note that the Kaplan–Meier curves anecdotally showed consistently longer survival time for those with complete resection. Numerous studies have shown that residual tumor burden following cytoreductive surgery, whether primary or IDS, is among the strongest negative prognostic factors for survival in patients with endometrial cancer [16,19,20,21,22]. Larger studies are critically needed to clarify the role of resection margin status on survival, particularly in patients diagnosed with advanced-stage disease.

The rate of complete tumor resection seen in our study was very similar to that seen in another cohort study, which evaluated patients who underwent PCS, NACT-IDS, or chemotherapy alone—both just slightly higher than 80% [22]. This is in contrast to the relatively low rate of 16% complete cytoreduction seen in a study done before the NACT-IDS approach had been established [22]. This stark difference supports the idea that NACT may be an advantageous approach for reducing tumor burden and improving the feasibility of complete resection in patients who likely would have been deemed ineligible for surgery in the past.

The RECIST criteria are a helpful radiographic framework for classifying the tumor response to NACT. In our study, both tumor response to NACT and resection margin status were associated with PFS in unadjusted analyses, suggesting that these factors may have prognostic relevance in patients undergoing NACT-IDS. These findings are consistent with other studies of patients with advanced-stage endometrial cancer. For example, one observational study of 42 patients who were ineligible for PCS found that a complete or partial RECIST response was associated with the likelihood of proceeding to IDS and having complete postoperative cytoreduction, which were in turn associated with improved survival [23]. Collectively, these data suggest that a radiographic response to NACT is clinically relevant for gauging disease progression and guiding surgical planning. However, the prognostic value of the tumor response to NACT may be closely linked to the extent of surgical cytoreduction, rather than functioning as an independent predictor of long-term outcomes, underscoring a potentially synergistic relationship of these factors within this treatment paradigm.

The 2020 World Health Organization updates recommended risk stratification approach for patients with advanced endometrial cancer is molecular classification [24]. Based on comprehensive genomic analyses conducted by The Cancer Genome Atlas project and subsequently validated in the Proactive Molecular Risk Classifier for Endometrial Cancer (ProMisE) and TransPORTEC studies, endometrial cancers are classified into 4 clinically significant molecular subgroups: POLE (DNA polymerase epsilon)-mutated, MMR-deficient, p53-abnormal, and no specific molecular profile (NSMP), the latter of which may be further stratified by estrogen receptor status. The prognostic value of molecular classification has been well studied in stage I through III endometrial cancer; however, findings supporting its prognostic value in advanced-stage endometrial cancer remain limited and findings have been mixed. One recent study evaluated survival among 363 patients with endometrial cancer stratified by the four molecular subgroups, including p53-abnormal, MMR-deficient, and estrogen receptor–positive and –negative NSMP [22]. The study showed that patients with MMR-deficient tumors had better overall survival than patients with p53-abnormal tumors, with comparative survival of 34 versus 21 months [22]. Conversely, two studies evaluating the role of molecular subtypes in patients with advanced endometrial cancer found no association between molecular classification and survival following PDS or NACT-IDS [13,25]. The studies evaluated patients with p53-abnormal, MMR-deficient, NSMP, and POLE-mutant subtypes, and the findings raise questions about the utility of molecular subtyping for prognoses in patients with advanced disease.

Our study included analyses of MMR and p53 status. While neither molecular marker was significantly associated with differences in survival, we did anecdotally observe that patients with MMR-deficient tumors showed slightly longer PFS, and this marker was associated with the NACT tumor response, in which most patients who had a complete or partial response had MMR-deficient tumors. Overall, our findings complement those from other studies regarding the prognostic value of molecular stratification, which showed mixed and sometimes conflicting results [13,22,25]. Our results suggest that patients with MMR-deficient tumors may have enhanced responsiveness to NACT, which could have prognostic implications, and that molecular class may be more relevant to the treatment response rather than overall survival outcomes. However, taken together with other studies, our results highlight the complexity of advanced disease and the need for controlled studies of NACT-IDS in advanced endometrial cancer. Importantly, molecular classification may influence not only prognosis but also the sequencing and intensity of multimodal therapy. As molecularly informed adjuvant treatment becomes increasingly integrated into clinical practice, future studies of NACT should evaluate whether molecular subtype predicts not only response to neoadjuvant chemotherapy but also benefit from postoperative chemotherapy, radiotherapy, immunotherapy, or combined treatment strategies.

## 5. Limitations

This study has several important limitations. First, its retrospective single-institution design and relatively small sample size limit statistical power and generalizability. Second, the study population was heterogeneous with respect to histologic subtype, molecular profile, disease burden, and systemic treatment received, reflecting real-world clinical practice but limiting the ability to identify subgroup-specific prognostic factors. The inclusion of patients treated over a prolonged study period also encompasses evolving systemic therapies, including the introduction of immune checkpoint inhibitors for selected patients.

Furthermore, this study was intended to be descriptive and exploratory rather than confirmatory. The analyses were hypothesis-generating and should not be interpreted as establishing causal relationships between clinicopathologic features and survival outcomes. Selection bias is also likely, as patients undergoing NACT-IDS were selected based on individual surgeons’ assessment of nonresectability or anticipated surgical morbidity rather than standardized criteria.

Additionally, treatment-related toxicity and postoperative functional recovery were not systematically captured and therefore could not be evaluated. These outcomes are particularly important in an older patient population receiving multimodal therapy and should be incorporated into future prospective studies.

## 6. Conclusions

This study provides a comprehensive clinicopathologic characterization of patients with advanced-stage endometrial cancer who underwent the alternative treatment approach of NACT for tumor burden reduction followed by IDS for tumor removal. Our findings suggest that having a molecular MMR-deficient subtype may, in part, be prognostic for a more favorable response to NACT, whereas p53 is not. Our analyses suggest that tumor response to NACT and postoperative tumor margin status may be helpful prognostic features for clinicians to consider. Our findings also reveal that 5-year survival outcomes are still very poor for this patient group, underscoring the need for more aggressive exploration of effective therapies for patients with advanced-stage disease. Overall, our study suggests that outcomes in advanced-stage endometrial cancer may involve a complex interplay between baseline tumor molecular features, responses to chemotherapy, and surgical factors.

## Figures and Tables

**Table 1:** Demographic and clinicopathologic characteristics of patients with advanced-stage endometrial cancer.

Characteristics	Result N = 42
Age at diagnosis, median (IQR), years	68 (63–72)
Race, n (%)	
Black	19 (45)
White	23 (55)
Body mass index, median (IQR), kg/m^2^	32.3 (27.6–38.3)
Histologic subtypes, n (%)	
Carcinosarcoma	3 (7.1)
Clear cell carcinoma	2 (4.8)
Endometrioid carcinoma	12 (28.6)
Mixed	3 (7.1)
Serous carcinoma	20 (47.6)
Undifferentiated	2 (4.8)
Tumor grade, n (%)	
Grade 1	5 (11.9)
Grade 2	4 (9.5)
Grade 3	33 (78.6)
Cancer stage at diagnosis, n (%)	
IIIB	1 (2.4)
IIIC	11 (26.2)
IVB	30 (71.4)
CA-125 at diagnosis, median (IQR), U/mL (n = 37)	117.0 (27–580)

Abbreviations: CA-125, cancer antigen 125; IQR, interquartile range.

**Table 2:** Pre-operative chemotherapeutic response, surgical features, surgical outcomes, and tumor molecular features of patients with advanced-stage endometrial cancer.

Characteristics	Result, n (%) N = 42
***Pre-operative treatment***	
^a^ NACT tumor response	
Complete response	5 (11.9)
Partial response	26 (61.9)
Stable disease	4 (9.5)
Progressive disease	7 (16.7)
***Surgical variables***	
Surgical approach	
Open	33 (78.6)
Robotic	9 (21.4)
Resection margin status	
R0—no residual tumor	35 (83.3)
R1—microscopic residual tumor	5 (11.9)
R2—macroscopic residual tumor	2 (4.8)
Lymphovascular space invasion	
Positive	22 (52.4)
Negative	20 (47.6)
***Tumor molecular features***	
Mismatch repair protein status (n = 29)	
Deficient	23 (79.3)
Proficient	6 (20.7)
p53 immunohistochemistry pattern (n = 31)	
Aberrant	16 (51.6)
Wild type	15 (48.4)

Abbreviations: NACT, neoadjuvant chemotherapy. ^a^ Responses per the RECIST (Response Evaluation Criteria in Solid Tumors) criteria [12] as determined through computed tomography (CT).

**Table 3:** Treatment and pathologic characteristics of patients with advanced-stage endometrial cancer who had complete or partial responses to preoperative neoadjuvant chemotherapy.

Treatment and Pathologic Characteristics by Tumor Response to NACT ^a^, n (%)		
	Complete Response n = 5	Partial Response n = 26
NACT cycles, n		
2 cycles	1 (20)	0 (0)
3 cycles	0 (0)	6 (23.1)
4 cycles	2 (40)	10 (38.5)
6 cycles	2 (40)	10 (38.5)
Additional neoadjuvant therapy, n (%)		
Bevacizumab	0	1 (3.8)
Trastuzumab	0	1 (3.8)
Dostarlimab	0	0
Adriamycin	1 (50)	0
Lymphovascular space invasion		
Positive	0	14 (53.8)
Negative	5 (100)	12 (46.2)
CR per final pathology analysis	2 (40)	1 (3.8)
Recurrence during follow-up	1 (20)	20 (76.9)

Abbreviations: NACT, neoadjuvant chemotherapy; IO, immunotherapy; n.a, not applicable; CR, complete response. ^a^ Responses per the RECIST (Response Evaluation Criteria in Solid Tumors) criteria [15] as determined through computed tomography (CT).

**Table 4:** Association of MMR status and P53 pattern with response to preoperative neoadjuvant chemotherapy in patients with advanced-stage endometrial cancer.

Preoperative Tumor Response to Neoadjuvant Chemotherapy ^a^, n (%) ^b^					
Molecular Features	CR	PR	SD	PD	*p*-Value ^c^
MMR protein status (N = 29)					0.013
Deficient (n = 23)	4 (17.4)	17 (73.9)	0 (0)	2 (8.7)	
Proficient (n = 6)	0 (0)	2 (33.3)	2 (33.3)	2 (33.3)	
p53 IHC pattern (N = 31)					0.365
Aberrant (n = 16)	1 (6.2)	13 (81.3)	1 (6.2)	1 (6.2)	
Wild type (n = 15)	3 (20.0)	8 (53.3)	1 (6.7)	3 (20.0)	

Abbreviations: CR, complete response; IHC, immunohistochemistry; MMR, mismatch repair; PD, progressive disease; PR, partial response; SD, stable disease. ^a^ Tumor response based on computed tomography (CT) according to the RECIST (Response Evaluation Criteria in Solid Tumors) criteria [15]. ^b^ Row percentages indicated. ^c^ Fisher’s exact test (Freeman-Halton extension).

**Table 5:** Univariate Cox analysis of clinical, surgical, and molecular factors associated with overall survival after neoadjuvant chemotherapy and interval debulking surgery for patients with advanced-stage endometrial cancer.

	Overall Survival Cox Regressions			Progression-Free Survival Cox Regressions		
Variables	Hazard Ratio	95% CI	*p*-Value	Hazard Ratio	95% CI	*p*-Value
Cancer stage at diagnosis *			0.22			0.11
IIIB	Reference			Reference		
IIIC	9.457 × 10^7^	[0, Inf]		7.18 × 10^7^	[0, Inf]	
IVB	7.892 × 10^7^	[0, Inf]		8.78 × 10^7^	[0, Inf]	
NACT tumor response			0.12			0.04
Complete response	Reference			Reference		
Partial response	4.410	[0.584, 33.3]		6.70	[0.896, 50.1]	
Stable disease	0.979	[0.061, 15.8]		2.18	[0.196, 24.2]	
Progressive disease	3.600	[0.397, 32.7]		5.20	[0.602, 44.9]	
Surgical approach			0.36			0.08
Open	Reference			Reference		
Robotic	0.64	[0.236, 1.73]		0.454	[0.173, 1.19]	
Resection margin status			0.06			0.04
R0	Reference			Reference		
R1/R2 **	2.66	[1.04, 6.82]				
Tumor molecular features						
MMR protein status (n = 29)			0.96			0.16
Deficient				Reference		
Proficient	0.963	[0.204, 4.54]		2.27	[0.755, 6.82]	
p53 IHC pattern (n = 31)			0.18			0.17
Aberrant	Reference			Reference		
Wild type	0.492	[0.172, 1.41]		0.547	[0.232, 1.29]	

* Model coefficients did not stabilize for either outcome due to small or zero cell counts. ** Note that while for OS the CI excluded 1, the overall likelihood ratio test exceeded 0.05. Abbreviations: IHC, immunohistochemistry; MMR, mismatch repair; NACT, neoadjuvant chemotherapy.

## Data Availability

All data generated or analyzed during this study are included in this published article. Individual patient data is only accessible to the research team per IRB protocol and is not public.
